# Supplementary material for: Quantitative MRI provides markers of intra-, inter-regional, and age-related differences in young adult cortical microstructure
Source: Neuroimage. 2018 Nov 15;182:429–40. doi: 10.1016/j.neuroimage.2017.11.066 (PMC6189523; doi:10.1016/j.neuroimage.2017.11.066)
Supplement: Supplementary [file mmc1.doc]

**Supplemental Methods**

**Image pre-processing and surface reconstruction**

**Image synthesis:** First, two synthetic FLASH volumes were created using the FreeSurfer mri_synthesize routine. Inputs to the routine were scaled quantitative PD and T_1_ (1/R_1_ volumes, with removal of a small number of negative and very high values produced by estimation errors). All MPM images were registered during the initial pre-processing stage in SPM (image generation with VBQ toolbox - see 2.3.1). Volumes later generated in FreeSurfer were therefore aligned to those input images (T1 and PD). For the first synthetic image, default FreeSurfer contrast parameters were specified. The second synthetic image was produced using the same PD and T1 input volumes, with contrast parameters specified (TR = 20 ms; α = 30°; TE = 2.5 ms). During synthesis, images were ‘conformed’ to 1mm^3^ isotropic resolution in FreeSurfer. Both synthetic images were then further scaled with AFNI 3dcalc; this additional linear scaling yielded image intensity properties closer to the optimal intensity values needed to segment tissue boundaries in FreeSurfer. The image synthesized with default contrast parameters was used as the main input to the FreeSurfer automated processing stream (following further pre-processing steps; see below). The image synthesized with specified contrast parameters was used at a later stage as input to the FreeSurfer Talairach transformation. Finally, a scaled and truncated version of the PD volume was produced with AFNI 3dcalc. This adjusted PD volume was used as input to the skull strip procedure (see below).

**Manual image adjustment:** Because the FreeSurfer reconstruction pipeline relies on highly homogeneous values within tissues, the FLASH image synthesized with default parameters was further adjusted for subjects within the first cohort (*n* = 34) using an in-house version of FreeSurfer (Csurf). Differences in the MPM acquisition led to improved image contrast in the second cohort (*n* = 59), and thus manual image adjustment was not required. Each subject’s synthetic image was hand-adjusted using a piecewise linear normalization procedure to linearly ramp intensity values of grey and white matter within isolated regions. Brightness values of voxels within the inferior and medial temporal lobes, temporal pole, long and short insular gyri, and ventro-medial pre-frontal cortex were gently rescaled (< 1.2x). Manual blink comparison between the synthetic volume and the labelled white matter surface was used to compare adjustments as each brightening iteration was applied. Care was taken to ensure that manual brightening did not cause grey and white matter to exceed the intensity value bounds specified for those tissue classes in FreeSurfer (grey matter: 50-70; white matter: 100-140). Manually brightened synthetic images were saved and used within the skull strip procedure.

**Skull strip**: Next, the subject’s adjusted quantitative PD volume (see image synthesis) was used as input to a customized skull strip procedure run in Csurf. Briefly, the skull strip procedure removed the skull and regions exterior to it from the image volume, rendering an image of remaining brain tissue (including cerebellum and brainstem). First, an elliptical surface (4th or 5th geodesic subtessellation of an icosahedron) was expanded from inside the PD volume, with expansion of the surface constrained by arrival at low intensity voxels (i.e., those containing CSF and/or the inner surface of the skull). The set of voxels intersecting the faces of the resulting surface was then flood-filled from the outside, thereby constraining the brain volume to the brighter voxels inside the surface region. Using this PD volume as a mask, flood-filled voxels in the volume were used to set the corresponding voxels in the subject’s default-parameter synthetic image to an intensity of zero. The boundaries of the flood-filled voxels within the skull-stripped PD image were then manually adjusted to correct for any local deviations into neural tissue (particularly in regions proximal to paranasal sinuses, prone to susceptibility artifacts). Manual adjustment involved reducing the intensity threshold for cortical grey matter (to a value of 40); the flood-filled boundary was then forced toward voxels below this threshold. Manual adjustment was applied to the synthetic volume; the skull-stripped synthetic volume was used as input to a custom version of the surface reconstruction pipeline.

**Surface reconstruction**: First, each subject’s skull-stripped synthetic volume was intensity normalized in FreeSurfer (using the mri_normalize routine). Normalized images were briefly inspected to ensure grey and white matter intensity values were within the appropriate ranges (white matter: 110; grey matter: 50-70). Next, the skull-stripped default parameter synthetic volume (see skull strip) was used to mask the contrast-specified synthetic volume (see image synthesis). This masked (i.e., skull-stripped), contrast-specified synthetic volume was then used as the input volume to an initial Talairach transformation process (run using the FreeSurfer mri_em_register routine). Next, a further normalization step was performed (using the -canorm parameter in FreeSurfer recon-all); the initial Talairach transform, the skull-stripped, default parameter synthetic volume and the intensity-normalized version of that volume were used as inputs. Following this, a multi-dimensional Talairach transformation was applied (using the -careg and -careginv parameters in recon-all); the normalized volume (produced by recon-all -canorm), the skull-stripped default parameter synthetic volume, and the initial Talairach transform were used as inputs. Finally, the full FreeSurfer recon-all pipeline was run for each subject (parameters specified can be found at: https://surfer.nmr.mgh.harvard.edu/fswiki/ReconAllDevTable; parameters used were those of the autorecon-2 stage, and the first 6 parameters from autorecon-3). Following reconstruction, pial and white matter surfaces were inspected blind to group (overlaid onto the normalised recon-all input volume), to identify local surface deviances. Surface for seven participants in Cohort 1 (4 violinists, 3 non-musicians) showed minor deviances such that the pial surface underestimated the true pial boundary; those participants’ synthetic volumes were re-brightened and re-run through the processes above. Re-inspection of these subjects’ surfaces indicated improved segmentations relative to the first attempt that were in line with the remainder of both cohorts.

Supplemental table 1: Multi-level model fit of ROI mean data - MT

| Terms | Coefficient | z | *p* | 95% CI Lower bound | 95% CI Upper bound |
| --- | --- | --- | --- | --- | --- |
| **Intercept** | 1.163852 | 61.02 | 0.00001 | 1.12647 | 1.201235 |
| **Depth** | -0.1514437 | -236.07 | 0.00001 | -0.152701 | -0.1501863 |
| **Depth x Depth** | 0.0232785 | 252.78 | 0.00001 | 0.023098 | 0.023459 |
| **Depth x Depth x Depth** | -0.0013631 | -224.3 | 0.00001 | -0.0013751 | -0.0013512 |
| **MPM cohort (ref: cohort 2)** | -0.0393915 | -8.46 | 0.00001 | -0.0485129 | -0.0302701 |
| **Gender (ref: male)** | 0.0041655 | 0.91 | 0.363 | -0.0048137 | 0.0131446 |
| **Age** | 0.0031525 | 3.97 | 0.00001 | 0.0015961 | 0.0047089 |
| **Age x Depth** | -0.0002147 | -7.94 | 0.00001 | -0.0002677 | -0.0001617 |
| **ROI** (ref: G_and_S_cingul_Mid_Post) |  |  |  |  |  |
| G_and_S_occipital_inf | -0.0090198 | -0.47 | 0.638 | -0.0466214 | 0.0285819 |
| G_and_S_subcentral | -0.0181594 | -0.95 | 0.344 | -0.0557611 | 0.0194422 |
| G_front_inf_Orbital | -0.0577246 | -3.01 | 0.003 | -0.0953263 | -0.020123 |
| G_front_sup | -0.0395833 | -2.06 | 0.039 | -0.0771849 | -0.0019816 |
| G_occipital_sup | -0.001184 | -0.06 | 0.951 | -0.0387856 | 0.0364177 |
| G_pariet_inf_Angular | -0.0279502 | -1.46 | 0.145 | -0.0655519 | 0.0096514 |
| G_parietal_sup | -0.0433116 | -2.26 | 0.024 | -0.0809132 | -0.0057099 |
| G_precuneus | -0.028817 | -1.5 | 0.133 | -0.0664186 | 0.0087847 |
| G_temp_sup_G_T_transv | 0.009343 | 0.49 | 0.626 | -0.0282586 | 0.0469447 |
| G_temp_sup_Plan_tempo | -0.0274095 | -1.43 | 0.153 | -0.0650111 | 0.0101921 |
| MT_thresh | -0.0113279 | -0.59 | 0.555 | -0.0489296 | 0.0262737 |
| S_collat_transv_post | 0.0106317 | 0.55 | 0.579 | -0.02697 | 0.0482333 |
| S_front_inf | -0.0379105 | -1.98 | 0.048 | -0.0755121 | -0.0003088 |
| S_front_middle | -0.0535731 | -2.79 | 0.005 | -0.0911748 | -0.0159715 |
| S_oc_middle_and_Lunatus | -0.0040505 | -0.21 | 0.833 | -0.0416522 | 0.0335511 |
| S_oc_sup_and_transversal | -0.0012721 | -0.07 | 0.947 | -0.0388738 | 0.0363295 |
| S_occipital_ant | -0.0172783 | -0.9 | 0.368 | -0.05488 | 0.0203233 |
| S_parieto_occipital | -0.0085082 | -0.44 | 0.657 | -0.0461098 | 0.0290935 |
| S_precentral_inf_part | -0.0312785 | -1.63 | 0.103 | -0.0688801 | 0.0063232 |
| S_precentral_sup_part | -0.0255282 | -1.33 | 0.183 | -0.0631298 | 0.0120735 |
| S_subparietal | -0.0136979 | -0.71 | 0.475 | -0.0512995 | 0.0239037 |
| V1_thresh | 0.0030433 | 0.16 | 0.874 | -0.0345584 | 0.0406449 |
| **ROI x Age** |  |  |  |  |  |
| G_and_S_occipital_inf | 0.0023626 | 2.85 | 0.004 | 0.0007369 | 0.0039884 |
| G_and_S_subcentral | 0.0013936 | 1.68 | 0.093 | -0.0002322 | 0.0030194 |
| G_front_inf_Orbital | 0.0012281 | 1.48 | 0.139 | -0.0003977 | 0.0028538 |
| G_front_sup | 0.0008597 | 1.04 | 0.3 | -0.000766 | 0.0024855 |
| G_occipital_sup | 0.0033086 | 3.99 | 0.00001 | 0.0016828 | 0.0049344 |
| G_pariet_inf_Angular | 0.001441 | 1.74 | 0.082 | -0.0001848 | 0.0030668 |
| G_parietal_sup | 0.0032801 | 3.95 | 0.00001 | 0.0016543 | 0.0049059 |
| G_precuneus | 0.0019278 | 2.32 | 0.02 | 0.000302 | 0.0035536 |
| G_temp_sup_G_T_transv | 0.0033346 | 4.02 | 0.00001 | 0.0017088 | 0.0049604 |
| G_temp_sup_Plan_tempo | 0.0021005 | 2.53 | 0.011 | 0.0004747 | 0.0037263 |
| MT_thresh | 0.0032762 | 3.95 | 0.00001 | 0.0016504 | 0.004902 |
| S_collat_transv_post | 0.0010644 | 1.28 | 0.199 | -0.0005614 | 0.0026902 |
| S_front_inf | 0.0007743 | 0.93 | 0.351 | -0.0008515 | 0.0024001 |
| S_front_middle | 0.0006195 | 0.75 | 0.455 | -0.0010062 | 0.0022453 |
| S_oc_middle_and_Lunatus | 0.0019544 | 2.36 | 0.018 | 0.0003286 | 0.0035802 |
| S_oc_sup_and_transversal | 0.0020931 | 2.52 | 0.012 | 0.0004673 | 0.0037189 |
| S_occipital_ant | 0.0020127 | 2.43 | 0.015 | 0.0003869 | 0.0036385 |
| S_parieto_occipital | 0.001883 | 2.27 | 0.023 | 0.0002572 | 0.0035088 |
| S_precentral_inf_part | 0.0004028 | 0.49 | 0.627 | -0.0012229 | 0.0020286 |
| S_precentral_sup_part | 0.0020516 | 2.47 | 0.013 | 0.0004259 | 0.0036774 |
| S_subparietal | 0.0005199 | 0.63 | 0.531 | -0.0011059 | 0.0021457 |
| V1_thresh | 0.0006021 | 0.73 | 0.468 | -0.0010237 | 0.0022279 |
| **ROI x Age x Depth** |  |  |  |  |  |
| G_and_S_occipital_inf | -0.0002382 | -9.54 | 0.00001 | -0.0002871 | -0.0001892 |
| G_and_S_subcentral | -0.0000846 | -3.39 | 0.001 | -0.0001335 | -0.0000357 |
| G_front_inf_Orbital | 0.0000149 | 0.6 | 0.55 | -0.000034 | 0.0000638 |
| G_front_sup | -0.0000113 | -0.45 | 0.65 | -0.0000602 | 0.0000376 |
| G_occipital_sup | -0.0003681 | -14.75 | 0.00001 | -0.000417 | -0.0003192 |
| G_pariet_inf_Angular | -0.0001155 | -4.63 | 0.00001 | -0.0001645 | -0.0000666 |
| G_parietal_sup | -0.000218 | -8.73 | 0.00001 | -0.0002669 | -0.0001691 |
| G_precuneus | -0.0001322 | -5.3 | 0.00001 | -0.0001811 | -0.0000833 |
| G_temp_sup_G_T_transv | -0.0002449 | -9.81 | 0.00001 | -0.0002938 | -0.000196 |
| G_temp_sup_Plan_tempo | -0.0001476 | -5.91 | 0.00001 | -0.0001965 | -0.0000987 |
| MT_thresh | -0.0003069 | -12.29 | 0.00001 | -0.0003558 | -0.0002579 |
| S_collat_transv_post | -0.0002358 | -9.45 | 0.00001 | -0.0002847 | -0.0001869 |
| S_front_inf | -5.09E-06 | -0.2 | 0.838 | -0.000054 | 0.0000438 |
| S_front_middle | 8.45E-06 | 0.34 | 0.735 | -0.0000405 | 0.0000574 |
| S_oc_middle_and_Lunatus | -0.0002671 | -10.7 | 0.00001 | -0.000316 | -0.0002182 |
| S_oc_sup_and_transversal | -0.0002749 | -11.01 | 0.00001 | -0.0003238 | -0.0002259 |
| S_occipital_ant | -0.0001672 | -6.7 | 0.00001 | -0.0002161 | -0.0001183 |
| S_parieto_occipital | -0.0002059 | -8.25 | 0.00001 | -0.0002548 | -0.000157 |
| S_precentral_inf_part | 0.0000537 | 2.15 | 0.031 | 4.81E-06 | 0.0001027 |
| S_precentral_sup_part | -0.0000981 | -3.93 | 0.00001 | -0.000147 | -0.0000491 |
| S_subparietal | -0.0000474 | -1.9 | 0.057 | -0.0000963 | 1.50E-06 |
| V1_thresh | -0.0000771 | -3.09 | 0.002 | -0.0001261 | -0.0000282 |
| **Random-effects Parameters** |  |  |  |  |  |
| **Subjects** |  |  |  |  |  |
| **Variance (intercept)** | 0.0004178 |  |  | 0.000308 | 0.0005668 |
| **ROI** |  |  |  |  |  |
| **Variance (Depth)** | 0.0000145 |  |  | 0.0000135 | 0.0000155 |
| **Variance (intercept)** | 0.0015658 |  |  | 0.0014548 | 0.0016851 |
| **Covariance (Depth, intercept)** | -0.0001244 |  |  | -0.0001341 | 0.0001147 |
| **Variance (Residual)** | 0.0001114 |  |  | 0.0001089 | 0.000114 |

Supplemental table 2: Multi-level model fit of ROI mean data - R_1_

| Terms | Coefficient | z | *p* | 95% CI Lower bound | 95% CI Upper bound |
| --- | --- | --- | --- | --- | --- |
| **Intercept** | 0.7453713 | 83.82 | 0.00001 | 0.7279433 | 0.7627993 |
| **Depth** | -0.0624354 | -235.32 | 0.00001 | -0.0629554 | -0.0619153 |
| **Depth x Depth** | 0.0098556 | 236.69 | 0.00001 | 0.009774 | 0.0099372 |
| **Depth x Depth x Depth** | -0.0005884 | -214.13 | 0.00001 | -0.0005938 | -0.000583 |
| **MPM cohort (ref: cohort 2)** | -0.0253559 | -10.06 | 0.00001 | -0.0302979 | -0.020414 |
| **Gender (ref: male)** | 0.0006534 | 0.26 | 0.792 | -0.0042115 | 0.0055182 |
| **Age** | 0.0009238 | 2.53 | 0.012 | 0.0002073 | 0.0016404 |
| **Age x Depth** | -0.0000679 | -6.5 | 0.00001 | -0.0000883 | -0.0000474 |
| **ROI** (ref: G_and_S_cingul_Mid_Post) |  |  |  |  |  |
| G_and_S_occipital_inf | -0.0069426 | -0.94 | 0.349 | -0.021479 | 0.0075939 |
| G_and_S_subcentral | 0.0028452 | 0.38 | 0.701 | -0.0116913 | 0.0173816 |
| G_front_inf_Orbital | -0.0068903 | -0.93 | 0.353 | -0.0214268 | 0.0076461 |
| G_front_sup | -0.0087425 | -1.18 | 0.238 | -0.023279 | 0.0057939 |
| G_occipital_sup | -0.0061457 | -0.83 | 0.407 | -0.0206821 | 0.0083908 |
| G_pariet_inf_Angular | -0.0230018 | -3.1 | 0.002 | -0.0375382 | -0.0084653 |
| G_parietal_sup | -0.0235413 | -3.17 | 0.002 | -0.0380777 | -0.0090048 |
| G_precuneus | -0.024317 | -3.28 | 0.001 | -0.0388535 | -0.0097806 |
| G_temp_sup_G_T_transv | 0.0067933 | 0.92 | 0.36 | -0.0077431 | 0.0213298 |
| G_temp_sup_Plan_tempo | -0.0164088 | -2.21 | 0.027 | -0.0309453 | -0.0018724 |
| MT_thresh | -0.0202415 | -2.73 | 0.006 | -0.034778 | -0.0057051 |
| S_collat_transv_post | -0.0050022 | -0.67 | 0.5 | -0.0195387 | 0.0095342 |
| S_front_inf | -0.0085122 | -1.15 | 0.251 | -0.0230487 | 0.0060242 |
| S_front_middle | -0.0101126 | -1.36 | 0.173 | -0.0246491 | 0.0044238 |
| S_oc_middle_and_Lunatus | -0.0125124 | -1.69 | 0.092 | -0.0270488 | 0.0020241 |
| S_oc_sup_and_transversal | -0.0126422 | -1.7 | 0.088 | -0.0271786 | 0.0018943 |
| S_occipital_ant | -0.0228455 | -3.08 | 0.002 | -0.0373819 | -0.008309 |
| S_parieto_occipital | -0.0173429 | -2.34 | 0.019 | -0.0318794 | -0.0028065 |
| S_precentral_inf_part | -0.0071262 | -0.96 | 0.337 | -0.0216626 | 0.0074103 |
| S_precentral_sup_part | -0.007855 | -1.06 | 0.29 | -0.0223915 | 0.0066814 |
| S_subparietal | -0.0207785 | -2.8 | 0.005 | -0.0353149 | -0.006242 |
| V1_thresh | 0.0089582 | 1.21 | 0.227 | -0.0055783 | 0.0234946 |
| **ROI x Age** |  |  |  |  |  |
| G_and_S_occipital_inf | 0.0013106 | 4.08 | 0.00001 | 0.0006816 | 0.0019395 |
| G_and_S_subcentral | 0.0005437 | 1.69 | 0.09 | -0.0000852 | 0.0011727 |
| G_front_inf_Orbital | 0.0004121 | 1.28 | 0.199 | -0.0002168 | 0.001041 |
| G_front_sup | 0.0005475 | 1.71 | 0.088 | -0.0000814 | 0.0011765 |
| G_occipital_sup | 0.0020392 | 6.35 | 0.00001 | 0.0014102 | 0.0026681 |
| G_pariet_inf_Angular | 0.0012333 | 3.84 | 0.00001 | 0.0006044 | 0.0018623 |
| G_parietal_sup | 0.0018028 | 5.62 | 0.00001 | 0.0011738 | 0.0024317 |
| G_precuneus | 0.0013286 | 4.14 | 0.00001 | 0.0006997 | 0.0019575 |
| G_temp_sup_G_T_transv | 0.0018388 | 5.73 | 0.00001 | 0.0012098 | 0.0024677 |
| G_temp_sup_Plan_tempo | 0.0016305 | 5.08 | 0.00001 | 0.0010016 | 0.0022594 |
| MT_thresh | 0.0023272 | 7.25 | 0.00001 | 0.0016983 | 0.0029561 |
| S_collat_transv_post | 0.0013348 | 4.16 | 0.00001 | 0.0007059 | 0.0019637 |
| S_front_inf | 0.0006358 | 1.98 | 0.048 | 6.82E-06 | 0.0012647 |
| S_front_middle | 0.0003655 | 1.14 | 0.255 | -0.0002634 | 0.0009944 |
| S_oc_middle_and_Lunatus | 0.0017372 | 5.41 | 0.00001 | 0.0011083 | 0.0023661 |
| S_oc_sup_and_transversal | 0.0018106 | 5.64 | 0.00001 | 0.0011817 | 0.0024396 |
| S_occipital_ant | 0.0017835 | 5.56 | 0.00001 | 0.0011546 | 0.0024124 |
| S_parieto_occipital | 0.0017666 | 5.51 | 0.00001 | 0.0011377 | 0.0023955 |
| S_precentral_inf_part | 0.0004356 | 1.36 | 0.175 | -0.0001934 | 0.0010645 |
| S_precentral_sup_part | 0.0010681 | 3.33 | 0.001 | 0.0004391 | 0.001697 |
| S_subparietal | 0.0008845 | 2.76 | 0.006 | 0.0002556 | 0.0015135 |
| V1_thresh | 0.001422 | 4.43 | 0.00001 | 0.0007931 | 0.002051 |
| **ROI x Age x Depth** |  |  |  |  |  |
| G_and_S_occipital_inf | -0.0000767 | -7.97 | 0.00001 | -0.0000956 | -0.0000579 |
| G_and_S_subcentral | -0.0000245 | -2.54 | 0.011 | -0.0000433 | -5.60E-06 |
| G_front_inf_Orbital | 0.0000221 | 2.3 | 0.022 | 3.24E-06 | 0.000041 |
| G_front_sup | 0.0000115 | 1.19 | 0.234 | -7.42E-06 | 0.0000303 |
| G_occipital_sup | -0.000123 | -12.78 | 0.00001 | -0.0001419 | -0.0001041 |
| G_pariet_inf_Angular | -0.0000294 | -3.05 | 0.002 | -0.0000483 | -0.0000105 |
| G_parietal_sup | -0.0000745 | -7.74 | 0.00001 | -0.0000934 | -0.0000557 |
| G_precuneus | -0.0000506 | -5.25 | 0.00001 | -0.0000694 | -0.0000317 |
| G_temp_sup_G_T_transv | -0.000072 | -7.47 | 0.00001 | -0.0000908 | -0.0000531 |
| G_temp_sup_Plan_tempo | -0.0000633 | -6.57 | 0.00001 | -0.0000822 | -0.0000444 |
| MT_thresh | -0.0001274 | -13.23 | 0.00001 | -0.0001463 | -0.0001086 |
| S_collat_transv_post | -0.0001024 | -10.63 | 0.00001 | -0.0001212 | -0.0000835 |
| S_front_inf | -0.0000129 | -1.34 | 0.18 | -0.0000318 | 5.95E-06 |
| S_front_middle | -1.28E-06 | -0.13 | 0.894 | -0.0000202 | 0.0000176 |
| S_oc_middle_and_Lunatus | -0.0001096 | -11.38 | 0.00001 | -0.0001285 | -0.0000908 |
| S_oc_sup_and_transversal | -0.0001142 | -11.86 | 0.00001 | -0.000133 | -0.0000953 |
| S_occipital_ant | -0.0000782 | -8.12 | 0.00001 | -0.0000971 | -0.0000593 |
| S_parieto_occipital | -0.0000943 | -9.79 | 0.00001 | -0.0001131 | -0.0000754 |
| S_precentral_inf_part | 5.28E-06 | 0.55 | 0.583 | -0.0000136 | 0.0000242 |
| S_precentral_sup_part | -0.0000376 | -3.91 | 0.00001 | -0.0000565 | -0.0000188 |
| S_subparietal | -0.0000376 | -3.9 | 0.00001 | -0.0000565 | -0.0000187 |
| V1_thresh | -0.0000635 | -6.6 | 0.00001 | -0.0000824 | -0.0000447 |
| **Random-effects Parameters** |  |  |  |  |  |
| **Subjects** |  |  |  |  |  |
| **Variance (intercept)** | 0.000126 |  |  | 0.0000936 | 0.0001695 |
| **ROI** |  |  |  |  |  |
| **Variance (Depth)** | 2.05E-06 |  |  | 1.91E-06 | 2.20E-06 |
| **Variance (intercept)** | 0.0002344 |  |  | 0.000216 | 0.0002542 |
| **Covariance (Depth, intercept)** | -0.0000183 |  |  | -0.0000198 | -0.0000167 |
| **Variance (Residual)** | 0.0000228 |  |  | 0.0000223 | 0.0000233 |

Supplemental table 3: Multi-level model fit of ROI mean data - R_2_*

| Terms | Coefficient | z | *p* | 95% CI Lower bound | 95% CI Upper bound |
| --- | --- | --- | --- | --- | --- |
| **Intercept** | 16.43493 | 36.5 | 0.00001 | 15.55233 | 17.31754 |
| **Depth** | -0.6213169 | -43.45 | 0.00001 | -0.649343 | -0.5932909 |
| **Depth x Depth** | 0.0436316 | 170.4 | 0.00001 | 0.0431298 | 0.0441335 |
| **MPM cohort (ref: cohort 2)** | 0.4453568 | 3.72 | 0.00001 | 0.2107049 | 0.6800088 |
| **Gender (ref: male)** | 0.0988899 | 0.84 | 0.401 | -0.1321037 | 0.3298835 |
| **Age** | 0.0531702 | 2.88 | 0.004 | 0.0169307 | 0.0894098 |
| **Age x Depth** | -0.0044006 | -5.75 | 0.00001 | -0.0059015 | -0.0028998 |
| **ROI** (ref: G_and_S_cingul_Mid_Post) |  |  |  |  |  |
| G_and_S_occipital_inf | 1.101212 | 2.59 | 0.009 | 0.2694713 | 1.932953 |
| G_and_S_subcentral | -0.0381351 | -0.09 | 0.928 | -0.8698759 | 0.7936056 |
| G_front_inf_Orbital | -1.032786 | -2.43 | 0.015 | -1.864527 | -0.2010451 |
| G_front_sup | -0.6631401 | -1.56 | 0.118 | -1.494881 | 0.1686007 |
| G_occipital_sup | 1.68779 | 3.98 | 0.00001 | 0.8560488 | 2.51953 |
| G_pariet_inf_Angular | -0.0093144 | -0.02 | 0.982 | -0.8410552 | 0.8224264 |
| G_parietal_sup | 0.2864955 | 0.68 | 0.5 | -0.5452453 | 1.118236 |
| G_precuneus | 0.0721609 | 0.17 | 0.865 | -0.7595799 | 0.9039017 |
| G_temp_sup_G_T_transv | -0.3094683 | -0.73 | 0.466 | -1.141209 | 0.5222725 |
| G_temp_sup_Plan_tempo | -0.1736039 | -0.41 | 0.682 | -1.005345 | 0.6581369 |
| MT_thresh | 0.9594316 | 2.26 | 0.024 | 0.1276908 | 1.791172 |
| S_collat_transv_post | 1.485449 | 3.5 | 0.00001 | 0.6537078 | 2.317189 |
| S_front_inf | -0.6940483 | -1.64 | 0.102 | -1.525789 | 0.1376925 |
| S_front_middle | -0.9451584 | -2.23 | 0.026 | -1.776899 | -0.1134176 |
| S_oc_middle_and_Lunatus | 1.224062 | 2.88 | 0.004 | 0.3923212 | 2.055803 |
| S_oc_sup_and_transversal | 1.556641 | 3.67 | 0.00001 | 0.7249006 | 2.388382 |
| S_occipital_ant | 0.3863204 | 0.91 | 0.363 | -0.4454204 | 1.218061 |
| S_parieto_occipital | 0.519481 | 1.22 | 0.221 | -0.3122598 | 1.351222 |
| S_precentral_inf_part | -0.3801453 | -0.9 | 0.37 | -1.211886 | 0.4515955 |
| S_precentral_sup_part | 0.1466262 | 0.35 | 0.73 | -0.6851146 | 0.978367 |
| S_subparietal | -0.6771363 | -1.6 | 0.111 | -1.508877 | 0.1546044 |
| V1_thresh | 2.474066 | 5.83 | 0.00001 | 1.642325 | 3.305807 |
| **ROI x Age** |  |  |  |  |  |
| G_and_S_occipital_inf | -0.0089581 | -0.5 | 0.619 | -0.0442929 | 0.0263767 |
| G_and_S_subcentral | 0.0109824 | 0.61 | 0.542 | -0.0243524 | 0.0463173 |
| G_front_inf_Orbital | 0.0253868 | 1.41 | 0.159 | -0.009948 | 0.0607217 |
| G_front_sup | 0.0242973 | 1.35 | 0.178 | -0.0110376 | 0.0596321 |
| G_occipital_sup | 0.0000525 | 0 | 0.998 | -0.0352824 | 0.0353873 |
| G_pariet_inf_Angular | 0.0273334 | 1.52 | 0.129 | -0.0080014 | 0.0626683 |
| G_parietal_sup | 0.037464 | 2.08 | 0.038 | 0.0021291 | 0.0727988 |
| G_precuneus | 0.031402 | 1.74 | 0.082 | -0.0039329 | 0.0667368 |
| G_temp_sup_G_T_transv | 0.0867253 | 4.81 | 0.00001 | 0.0513905 | 0.1220601 |
| G_temp_sup_Plan_tempo | 0.0430388 | 2.39 | 0.017 | 0.0077039 | 0.0783736 |
| MT_thresh | 0.0183895 | 1.02 | 0.308 | -0.0169454 | 0.0537243 |
| S_collat_transv_post | -0.0228996 | -1.27 | 0.204 | -0.0582344 | 0.0124353 |
| S_front_inf | 0.024883 | 1.38 | 0.168 | -0.0104519 | 0.0602178 |
| S_front_middle | 0.0090242 | 0.5 | 0.617 | -0.0263106 | 0.0443591 |
| S_oc_middle_and_Lunatus | -0.005106 | -0.28 | 0.777 | -0.0404408 | 0.0302289 |
| S_oc_sup_and_transversal | -0.0095752 | -0.53 | 0.595 | -0.0449101 | 0.0257596 |
| S_occipital_ant | 0.0196818 | 1.09 | 0.275 | -0.015653 | 0.0550166 |
| S_parieto_occipital | 0.0259549 | 1.44 | 0.15 | -0.00938 | 0.0612897 |
| S_precentral_inf_part | 0.0219557 | 1.22 | 0.223 | -0.0133792 | 0.0572905 |
| S_precentral_sup_part | 0.0277002 | 1.54 | 0.124 | -0.0076346 | 0.0630351 |
| S_subparietal | 0.0426677 | 2.37 | 0.018 | 0.0073329 | 0.0780025 |
| V1_thresh | -0.0452831 | -2.51 | 0.012 | -0.080618 | -0.0099483 |
| **ROI x Age x Depth** |  |  |  |  |  |
| G_and_S_occipital_inf | 0.0040669 | 5.75 | 0.00001 | 0.0026818 | 0.0054519 |
| G_and_S_subcentral | 0.0010081 | 1.43 | 0.154 | -0.000377 | 0.0023932 |
| G_front_inf_Orbital | -0.001223 | -1.73 | 0.084 | -0.0026081 | 0.0001621 |
| G_front_sup | -0.0011259 | -1.59 | 0.111 | -0.002511 | 0.0002592 |
| G_occipital_sup | 0.0031355 | 4.44 | 0.00001 | 0.0017504 | 0.0045206 |
| G_pariet_inf_Angular | 0.0011924 | 1.69 | 0.092 | -0.0001927 | 0.0025775 |
| G_parietal_sup | -0.0001675 | -0.24 | 0.813 | -0.0015526 | 0.0012176 |
| G_precuneus | 0.0012991 | 1.84 | 0.066 | -0.000086 | 0.0026842 |
| G_temp_sup_G_T_transv | -0.0033267 | -4.71 | 0.00001 | -0.0047118 | -0.0019416 |
| G_temp_sup_Plan_tempo | -0.0011066 | -1.57 | 0.117 | -0.0024917 | 0.0002784 |
| MT_thresh | 0.0006587 | 0.93 | 0.351 | -0.0007264 | 0.0020438 |
| S_collat_transv_post | 0.0041653 | 5.89 | 0.00001 | 0.0027802 | 0.0055503 |
| S_front_inf | 0.0001832 | 0.26 | 0.795 | -0.0012019 | 0.0015683 |
| S_front_middle | 0.0005671 | 0.8 | 0.422 | -0.000818 | 0.0019522 |
| S_oc_middle_and_Lunatus | 0.0050314 | 7.12 | 0.00001 | 0.0036463 | 0.0064164 |
| S_oc_sup_and_transversal | 0.003195 | 4.52 | 0.00001 | 0.0018099 | 0.0045801 |
| S_occipital_ant | 0.0015623 | 2.21 | 0.027 | 0.0001772 | 0.0029473 |
| S_parieto_occipital | 0.0019321 | 2.73 | 0.006 | 0.0005471 | 0.0033172 |
| S_precentral_inf_part | 0.0001114 | 0.16 | 0.875 | -0.0012737 | 0.0014964 |
| S_precentral_sup_part | -0.0005311 | -0.75 | 0.452 | -0.0019162 | 0.000854 |
| S_subparietal | 0.0021725 | 3.07 | 0.002 | 0.0007874 | 0.0035576 |
| V1_thresh | 0.0095083 | 13.45 | 0.00001 | 0.0081233 | 0.0108934 |
| **Random-effects Parameters** |  |  |  |  |  |
| **Subjects** |  |  |  |  |  |
| **Variance (intercept)** | 0.2804993 |  |  | 0.2076692 | 0.3788712 |
| **ROI** |  |  |  |  |  |
| **Variance (Depth)** | 0.0123715 |  |  | 0.0116076 | 0.0131858 |
| **Variance (intercept)** | 0.4673403 |  |  | 0.4297264 | 0.5082467 |
| **Covariance (Depth, intercept)** | -0.051425 |  |  | -0.0565685 | -0.0462815 |
| **Variance (Residual)** | 0.0427286 |  |  | 0.0417665 | 0.043713 |

Supplemental table 4: Multi-level model fit of ROI mean data - PD*

| Terms | Coefficient | z | *p* | 95% CI Lower bound | 95% CI Upper bound |
| --- | --- | --- | --- | --- | --- |
| **Intercept** | 78.52895 | 100.32 | 0.00001 | 76.9947 | 80.06321 |
| **Depth** | 2.748519 | 171.59 | 0.00001 | 2.717124 | 2.779915 |
| **Depth x Depth** | -0.3866793 | -184.17 | 0.00001 | -0.3907944 | -0.3825642 |
| **Depth x Depth x Depth** | 0.0212191 | 153.15 | 0.00001 | 0.0209475 | 0.0214907 |
| **MPM cohort (ref: cohort 2)** | 0.2669198 | 1.17 | 0.241 | -0.1791553 | 0.7129948 |
| **Gender (ref: male)** | 0.1028447 | 0.46 | 0.646 | -0.3362759 | 0.5419653 |
| **Age** | -0.0398941 | -1.25 | 0.21 | -0.1023089 | 0.0225206 |
| **Age x Depth** | 0.0036487 | 5.13 | 0.00001 | 0.0022535 | 0.0050438 |
| **ROI** (ref: G_and_S_cingul_Mid_Post) |  |  |  |  |  |
| G_and_S_occipital_inf | -0.533053 | -0.82 | 0.41 | -1.800068 | 0.7339615 |
| G_and_S_subcentral | -1.392181 | -2.15 | 0.031 | -2.659196 | -0.125167 |
| G_front_inf_Orbital | -0.7960699 | -1.23 | 0.218 | -2.063084 | 0.4709446 |
| G_front_sup | -0.8648037 | -1.34 | 0.181 | -2.131818 | 0.4022108 |
| G_occipital_sup | -2.410334 | -3.73 | 0.00001 | -3.677349 | -1.14332 |
| G_pariet_inf_Angular | -1.670115 | -2.58 | 0.01 | -2.937129 | -0.4031004 |
| G_parietal_sup | -1.147287 | -1.77 | 0.076 | -2.414301 | 0.119728 |
| G_precuneus | -1.465618 | -2.27 | 0.023 | -2.732633 | -0.198604 |
| G_temp_sup_G_T_transv | -1.168833 | -1.81 | 0.071 | -2.435847 | 0.0981819 |
| G_temp_sup_Plan_tempo | -1.262658 | -1.95 | 0.051 | -2.529672 | 0.0043566 |
| MT_thresh | -0.5569336 | -0.86 | 0.389 | -1.823948 | 0.7100809 |
| S_collat_transv_post | 0.4455563 | 0.69 | 0.491 | -0.8214582 | 1.712571 |
| S_front_inf | -0.4258784 | -0.66 | 0.51 | -1.692893 | 0.841136 |
| S_front_middle | -0.7565988 | -1.17 | 0.242 | -2.023613 | 0.5104157 |
| S_oc_middle_and_Lunatus | 0.1132813 | 0.18 | 0.861 | -1.153733 | 1.380296 |
| S_oc_sup_and_transversal | -0.2514439 | -0.39 | 0.697 | -1.518458 | 1.015571 |
| S_occipital_ant | -0.359142 | -0.56 | 0.579 | -1.626156 | 0.9078725 |
| S_parieto_occipital | -0.1265337 | -0.2 | 0.845 | -1.393548 | 1.140481 |
| S_precentral_inf_part | -0.2639472 | -0.41 | 0.683 | -1.530962 | 1.003067 |
| S_precentral_sup_part | -0.1185033 | -0.18 | 0.855 | -1.385518 | 1.148511 |
| S_subparietal | -0.9497052 | -1.47 | 0.142 | -2.21672 | 0.3173092 |
| V1_thresh | -0.6328061 | -0.98 | 0.328 | -1.899821 | 0.6342084 |
| **ROI x Age** |  |  |  |  |  |
| G_and_S_occipital_inf | -0.0213658 | -0.78 | 0.433 | -0.0748136 | 0.0320821 |
| G_and_S_subcentral | 0.0237397 | 0.87 | 0.384 | -0.0297082 | 0.0771875 |
| G_front_inf_Orbital | -0.0269249 | -0.99 | 0.323 | -0.0803728 | 0.0265229 |
| G_front_sup | -0.0048218 | -0.18 | 0.86 | -0.0582696 | 0.0486261 |
| G_occipital_sup | 0.0609001 | 2.23 | 0.026 | 0.0074522 | 0.1143479 |
| G_pariet_inf_Angular | 0.0210201 | 0.77 | 0.441 | -0.0324278 | 0.0744679 |
| G_parietal_sup | -0.000348 | -0.01 | 0.99 | -0.0537959 | 0.0530998 |
| G_precuneus | 0.018602 | 0.68 | 0.495 | -0.0348459 | 0.0720498 |
| G_temp_sup_G_T_transv | -0.0500003 | -1.83 | 0.067 | -0.1034482 | 0.0034475 |
| G_temp_sup_Plan_tempo | -0.012135 | -0.44 | 0.656 | -0.0655828 | 0.0413129 |
| MT_thresh | -0.0523068 | -1.92 | 0.055 | -0.1057547 | 0.001141 |
| S_collat_transv_post | -0.0249784 | -0.92 | 0.36 | -0.0784262 | 0.0284695 |
| S_front_inf | 0.0042419 | 0.16 | 0.876 | -0.0492059 | 0.0576898 |
| S_front_middle | 0.0249307 | 0.91 | 0.361 | -0.0285171 | 0.0783786 |
| S_oc_middle_and_Lunatus | -0.0152124 | -0.56 | 0.577 | -0.0686602 | 0.0382355 |
| S_oc_sup_and_transversal | -0.0158683 | -0.58 | 0.561 | -0.0693162 | 0.0375795 |
| S_occipital_ant | -0.0203857 | -0.75 | 0.455 | -0.0738336 | 0.0330621 |
| S_parieto_occipital | -0.0086396 | -0.32 | 0.751 | -0.0620875 | 0.0448082 |
| S_precentral_inf_part | 0.0124009 | 0.45 | 0.649 | -0.041047 | 0.0658487 |
| S_precentral_sup_part | -0.006002 | -0.22 | 0.826 | -0.0594498 | 0.0474459 |
| S_subparietal | 0.0240228 | 0.88 | 0.378 | -0.029425 | 0.0774707 |
| V1_thresh | 0.0401446 | 1.47 | 0.141 | -0.0133032 | 0.0935925 |
| **ROI x Age x Depth** |  |  |  |  |  |
| G_and_S_occipital_inf | -0.0005331 | -0.81 | 0.417 | -0.0018207 | 0.0007544 |
| G_and_S_subcentral | 0.0007332 | 1.12 | 0.264 | -0.0005543 | 0.0020208 |
| G_front_inf_Orbital | -0.0013289 | -2.02 | 0.043 | -0.0026165 | -0.0000414 |
| G_front_sup | -0.0014712 | -2.24 | 0.025 | -0.0027588 | -0.0001837 |
| G_occipital_sup | 0.000356 | 0.54 | 0.588 | -0.0009315 | 0.0016436 |
| G_pariet_inf_Angular | -0.0001387 | -0.21 | 0.833 | -0.0014263 | 0.0011488 |
| G_parietal_sup | 0.000497 | 0.76 | 0.449 | -0.0007906 | 0.0017846 |
| G_precuneus | -0.0006511 | -0.99 | 0.322 | -0.0019386 | 0.0006365 |
| G_temp_sup_G_T_transv | 0.0044794 | 6.82 | 0.00001 | 0.0031918 | 0.0057669 |
| G_temp_sup_Plan_tempo | 0.0012467 | 1.9 | 0.058 | -0.0000409 | 0.0025342 |
| MT_thresh | 0.0037955 | 5.78 | 0.00001 | 0.0025079 | 0.005083 |
| S_collat_transv_post | 0.0003402 | 0.52 | 0.605 | -0.0009473 | 0.0016278 |
| S_front_inf | -0.0021902 | -3.33 | 0.001 | -0.0034778 | -0.0009027 |
| S_front_middle | -0.0038877 | -5.92 | 0.00001 | -0.0051752 | -0.0026001 |
| S_oc_middle_and_Lunatus | -0.0006958 | -1.06 | 0.29 | -0.0019833 | 0.0005918 |
| S_oc_sup_and_transversal | 0.0001589 | 0.24 | 0.809 | -0.0011287 | 0.0014465 |
| S_occipital_ant | 0.0000189 | 0.03 | 0.977 | -0.0012687 | 0.0013064 |
| S_parieto_occipital | 0.0015523 | 2.36 | 0.018 | 0.0002647 | 0.0028399 |
| S_precentral_inf_part | -0.0016743 | -2.55 | 0.011 | -0.0029619 | -0.0003867 |
| S_precentral_sup_part | 0.0003374 | 0.51 | 0.607 | -0.0009501 | 0.001625 |
| S_subparietal | -0.0018907 | -2.88 | 0.004 | -0.0031782 | -0.0006031 |
| V1_thresh | -0.006448 | -9.82 | 0.00001 | -0.0077355 | -0.0051604 |
| **Random-effects Parameters** |  |  |  |  |  |
| **Subjects** |  |  |  |  |  |
| **Variance (intercept)** | 1.02823 |  |  | 0.7624551 | 1.386647 |
| **ROI** |  |  |  |  |  |
| **Variance (Depth)** | 0.0103409 |  |  | 0.0096813 | 0.0110455 |
| **Variance (intercept)** | 0.8291677 |  |  | 0.7667966 | 0.8966121 |
| **Covariance (Depth, intercept)** | -0.0493045 |  |  | -0.0556379 | -0.0429712 |
| **Variance (Residual)** | 0.0579096 |  |  | 0.0566056 | 0.0592436 |

**Figure Captions**
